# Supplementary material for: Use of Melatonin and/on Ramelteon for the Treatment of Insomnia in Older Adults: A Systematic Review and Meta-Analysis
Source: J Clin Med. 2022 Aug 31;11(17):5138. doi: 10.3390/jcm11175138 (PMC9456584; doi:10.3390/jcm11175138)
Supplement: Supplementary file 1 [file jcm-11-05138-s001.zip › jcm-1850971-supplementary.pdf]

Supplementary material:

Figure S1: Subjective Sleep Outcomes.

Model	Study	Total N	Statistics for each study: Std diff in means	Upper limit	Lower limit	Z-Value	p-Value
	**TOTAL SLEEP TIME**						
	Andrade 1999; 5.4 mg	33	0.71	0.01	1.42	1.97	0.05
	Baskett 2001; 5.0 mg	40	-4.82	-6.05	-3.60	-7.72	0.00
	Dobkin 2006; 8.0 mg*	40	1.63	0.92	2.35	4.47	0.00
	Roth 2007; 4.0 mg*	200	0.06	-0.22	0.34	0.41	0.68
	Roth 2007; 8.0 mg*	200	0.47	0.19	0.76	3.31	0.00
	Wade 2010; 2.0 mg	281	0.21	-0.02	0.45	1.79	0.07
	Richardson 2009; 8.0mg*	205	0.44	0.17	0.72	3.14	0.00
	Roth 2006; 4.0 mg*	555	0.14	-0.03	0.30	1.63	0.10
	Roth 2006; 8.0 mg*	548	0.08	-0.09	0.25	0.94	0.35
Random	Overall		0.15	-0.18	0.48	0.89	0.38
	Heterogeneity I^2=91.3%						
	**SLEEP LATENCY**						
	Andrade 1999; 5.4 mg	33	1.02	0.29	1.75	2.74	0.01
	Baskett 2001; 5.0 mg	34	14.02	10.40	17.64	7.59	0.00
	Dobkin 2006; 8.0 mg	20	1.26	0.59	1.94	3.65	0.00
	Wade 2010; 2.0 mg	281	0.37	0.14	0.61	3.09	0.00
	Wade 2007; 2.0 mg	334	0.07	-0.14	0.29	0.68	0.50
	Mini 2007; 8.0 mg	327	0.37	0.15	0.59	3.29	0.00
	Richardson 2009; 8.0 mg*	248	0.32	0.05	0.60	2.31	0.02
	Roth 2006; 4.0 mg*	829	0.19	0.02	0.35	2.20	0.03
	Roth 2006; 8.0 mg*	829	0.22	0.05	0.39	2.58	0.01
	Roth 2007; 4.0 mg*	100	0.22	-0.06	0.50	1.56	0.12
	Roth 2007; 8.0 mg*	100	0.16	-0.12	0.44	1.15	0.25
Random	Overall		0.40	0.17	0.63	3.39	0.00
	Heterogeneity I^2=86.6%						

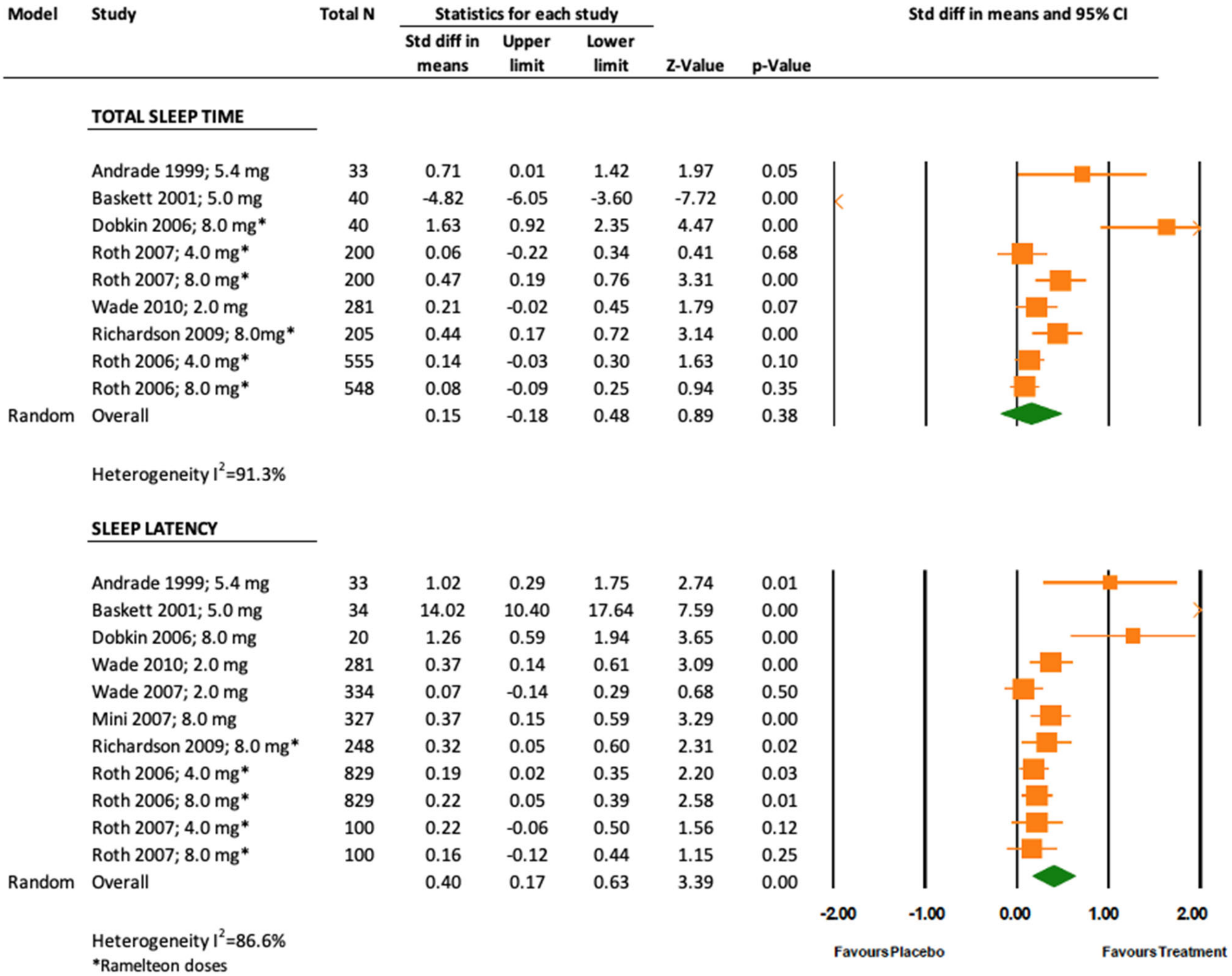

*Ramelteon doses

Table S1: sleep quality outcomes.

Study Author, Year	Outcome Measure	Sleep Quality	
		**Treatment**	**Placebo**
**Melatonin**			
Almeida et al, 2003[23]	Sleep logs with analogue visual scale	On a scale of 0 (poor)-5 (excellent)<br>0.3mg: 2.4 ± 1.1<br>1.0mg: 2.2 ± 1.6	2.0 ± 1.3
Baskett et al, 2001[25]	PSQI	PSQI Scores: 10 (8-11)	4 (3-5)
Dobkin et al, 2006[26]	Sleep diaries and self-report questionnaires	Significant improvements were observed in patient-reported sleep quality (10-point Likert scale) p<.001	
Fainstein et al,1997[27]	Sleep logs with 0-10 scale	Sleep quality improved on 3mg/day dose of melatonin for 21 days on a scale of 0-10	
Lemoine et al, 2007[31]	LSEQ, sleep diaries	Change in subjective QOS; -22.5	Change in subjective QOS; -16.5
Lemoine et al, 2011[32]	Sleep diaries	The mean number of nights by which patients reported sleep quality as "good" or "very good" was significantly higher during intervention than before treatment p<0.001	
Rondanelli et al, 2011[37]	PSQI; LSEQ, Wearable Armband-Shaped Sensor	PSQI scores: 5.5 ± 1.9	PSQI scores:<br>12.0 ± 4.4
Wade et al, 2010[42]	Sleep diary, PSQI	Change in subjective sleep quality from baseline:<br>-0.2 ± 0.56<br>Change in PSQI from baseline:<br>-1.86 ± 2.93	Change in subjective sleep quality from baseline:<br>-0.12 ± 0.57<br><br>Change in PSQI from baseline:<br>1.19 ± 2.53
Wade et al, 2007[41]	PSQI, LSEQ, sleep diary	LSEQ 45.9 ± 16.0<br>PSQI 8.1 ± 3.7<br>Sleep diary subjective QOS 3.0 ± 0.8	LSEQ 49.5 ± 14.8<br>PSQI 8.6 ± 3.7<br>Sleep diary

			subjective QOS 2.9 ± 0.9
**Ramelteon**			
Roth et al, 2007[39]	Polysomnography; Post Sleep Questionnaire	4.0mg: 3.7 (1.0)<br>8.0mg: 3.8 (1.0);<br>Sleep quality was evaluated on a 7-point scale Likert scale with 1 = excellent and 7 = extremely poor	3.8 (1.0)

LSEQ-Leeds Sleep Evaluation Questionnaire, PSQI-Pittsburgh Sleep Quality Index, PSG-Polysomnography, QOS-quality of sleep
